# pECGreview: Assessment of a Novel Tool to Evaluate the Accuracy of Pediatric ECG Interpretation Skills

**DOI:** 10.1007/s00246-024-03556-z

**Published:** 2024-07-02

**Authors:** Xander Jacquemyn, Karine Guerrier, Evan Harvey, Sean Tackett, Shelby Kutty, Glenn T. Wetzel

**Affiliations:** 1 Helen B. Taussig Heart Center, Department of Pediatrics, Johns Hopkins Hospital, M2315, 1800 Orleans St, Baltimore, MD 21287 USA; 2 Department of Cardiovascular Sciences, KU Leuven, Leuven, Belgium; 3 Division of Peds Cardiology, Department of Pediatrics, University of Tennessee Health Science Center, College of Medicine, Memphis, TN USA; 4 Biostatistics, Epidemiology, and Data Management Core, Johns Hopkins School of Medicine, Baltimore, MD USA

**Keywords:** Electrocardiogram, Interpretation, Medical Education, Pediatric Cardiology

## Abstract

The skill of interpretation of the electrocardiogram (ECG) remains poor despite existing educational initiatives. We sought to evaluate the validity of using a subjective scoring system to assess the accuracy of ECG interpretations submitted by pediatric cardiology fellows, trainees, and faculty to the Pediatric ECG Review (pECGreview), a web-based ECG interpretation training program. We conducted a retrospective, cross-sectional study of responses submitted to pECGreview. ECG interpretations were assessed independently by four individuals with a range of experience. Accuracy was assessed using a 3-point scale: 100% for generally correct interpretations, 50% for over- or underdiagnosis of minor ECG abnormalities, and 0% for over- or underdiagnosis of major ECG abnormalities. Inter-rater agreement was assessed using expanded Bland–Altman plots, Pearson correlation coefficients, and Intraclass Correlation Coefficients (ICC). 1460 ECG interpretations by 192 participants were analyzed. 107 participants interpreted at least five ECGs. The mean accuracy score was 76.6 ± 13.7%. Participants were correct in 66.1 ± 5.1%, had minor over- or underdiagnosis in 21.5 ± 4.6% and major over- or underdiagnosis in 12.3 ± 3.9% of interpretations. Validation of agreement between evaluators demonstrated limits of agreement of 11.3%. Inter-rater agreement exhibited consistent patterns (all correlations ≥ 0.75). Absolute agreement was 0.74 (95% CI 0.69–0.80), and average measures agreement was 0.92 (95% CI 0.89–0.94). Accuracy score analysis of as few as five ECG interpretations submitted to pECGreview yielded good inter-rater reliability for assessing and ranking ECG interpretation skills in pediatric cardiology fellows in training.

## Introduction

The pediatric electrocardiogram (pECG) is a widely used diagnostic tool for cardiovascular symptoms in infants, children, and adolescents [1, 2]. Expertise in ECG interpretation, with a particular emphasis on dysrhythmias, can be critical to disease evaluation and management. Despite the necessity for competency at pECG interpretation, it has been previously identified as an area in need of enhancement [1–4]. A recent systematic review and meta-analysis reported that physicians at all training levels may have deficiencies in ECG competency, even after educational interventions [5]. Despite some reports of increased accuracy with progressive training and specialization, they demonstrated that even cardiologists had performance gaps [5]. The lack of competence poses a risk of misdiagnosis, potential overtreatment, and adverse outcomes burdening both patients and their families. Traditionally, efforts to improve competency have focused on interpreting a target number of ECGs during training [6]. However, there is little evidence to support this approach.

Multiple supplemental ECG interpretation training programs and interventions have been developed and studied in a variety of contexts. Even an interactive, social media-based approach has been described [7]. Despite these initiatives, there remains a paucity of literature that details the pECG interpretation skills and optimum training approaches for pediatric cardiology fellows, other trainees, and faculty.

Due to the complexity of ECG interpretation, various methodologies have been used to assess the quality of those interpretations. Validation of these methods has been limited by (1) the expense and time commitment needed to perform large scale studies, and (2) the qualitative nature of free-text ECG interpretations and variations in nomenclature.

To address the issues with pECG competency in pediatric cardiology fellows, Pediatric ECG Review (pECGreview), a web-based, ECG-of-the-week style program was developed as a supplemental training initiative. While this program now provides a widely available tool for training in ECG interpretation, it has also generated the first reported large-scale database of repeated pECG interpretations by physicians at a variety of training stages.

The aims of this cross-sectional study were (1) to evaluate the use of a subjective scoring system for assessing the accuracy of pECG interpretations by pediatric cardiology fellows, trainees, and faculty, and (2) to evaluate the feasibility of using pECGreview-derived data to assess the relative accuracy of ECG interpretation of individual participants in pECGreview.

## Materials and Methods

### Pediatric ECG Review Design

The pECGreview is a web-based, ECG-of-the-week style program hosted on a secure REDCap (Research Electronic Data Capture) platform [8]. ECGs are selected to provide a broad exposure to pECG diagnoses over the course of a 3-year curriculum. The program is designed to improve pECG reading skills by encouraging participants to commit to a specific interpretation of each week’s pECG (provided by a link sent by email). Participants subsequently receive a report on the “official” interpretation as compared to their assessment. Additional comments are provided for specific teaching points. Since its inception in April of 2019, 31 pediatric cardiology fellowship training centers and 630 participants have enrolled. The majority of participants are enrolled as part of a pECG reading group with a local facilitator who is available to provide feedback for individual questions.

Each ECG is presented with a brief clinical history of the patient. Participants provide free-text responses reflecting their final interpretation as well as any comments that they wish to provide, e.g., a differential diagnosis or treatment considerations. A “Severity” score is also a required component of the interpretation, indicating the level of concern (normal/minor observation/borderline vs abnormal vs urgent/requiring immediate follow-up).

### Study Design

The study design consists of a retrospective, cross-sectional review of de-identified responses to the pECGreview program (https://www.hopkinsmedicine.org/heart-vascular-institute/specialty-areas/pediatric-and-congenital-heart-center/research/our-research/pediatric-ecg-review). The study has been approved by The University of Tennessee Health Science Center Institutional Review Board and the Johns Hopkins Medicine Institutional Review Board. Response data from 19 ECGs interpreted between 1/29/22 and 6/25/22 were collected. Incomplete submissions or tracings that included pacemaker analysis or electrophysiologic study images were excluded. Participant demographic information, including level of training, specialty, and year in training, was obtained from data collected at the time of enrollment in pECGreview.

### Assessment of the Accuracy of Interpretations

Each of the ECGs in pECGreview has been assigned an “official” interpretation by an experienced pediatric electrophysiologist. That interpretation is shared with each participant in pECGreview after they have submitted their own independent free-text interpretation.

For the purpose of this study, a panel of four blinded, independent evaluators (XJ, KG, EH, and GW) has each assigned a subjective “Accuracy” score to each ECG interpretation submitted by pECGreview participants. This is done by comparing the submitted response to the benchmark “official” interpretation provided by pECGreview. The 3-point Accuracy scale value assigned was 100% for generally correct responses, 50% for the over or under diagnosis of a minor ECG abnormality, or 0% for the over or under diagnosis of a major ECG abnormality. The panel of evaluators was purposely selected to include members at various levels of training and experience to provide an assessment the robustness of the accuracy scoring system. Two evaluators are practicing pediatric electrophysiologists, one was a pediatric cardiology fellow in training, and one was a medical student in training.

Participants who submitted at least five ECG interpretations were also included in an analysis of the relative accuracy of each individual participant. For each of the four independent evaluators, the mean and standard deviation of the Accuracy scores for each pECGreview participant was calculated and presented in rank order. Mean scores were determined for each of the individual ECGs.

### Statistical Analysis

For comparison between scores of the four evaluators, an extended Bland–Altman plot was used [9] For those plots, the *x*-axis corresponds to the classical Bland–Altman plot interpretation, while the *y*-axis presents the intra-subject standard deviation instead of the observed differences, in accordance with suggestions proposed by Bland and Altman. In addition, 95% limits of agreement (LOAs) are calculated along with a 95% bias-corrected LOA by applying 1000 bootstraps. Furthermore, the mean absolute error (MAE) was calculated with Evaluator 1 as reference, and visually plotted. To explore pairwise inter-rater agreement, we calculated the Pearson correlation coefficient (*r*) between all Evaluators. To assess reliability, we computed the Intraclass Correlation Coefficient (ICC) and its variations: ICC(1), ICC(1k), ICC(2), and ICC(2k). ICC(1) gauges single-rater consistency and is calculated using one-way analysis of variance (ANOVA) fixed effects model: [(Mean sum of Squares Between the participants (MSB)−Mean sum of Squares Within the participants (MSW))/(MSB + (Evaluators-1)×MSW)]. ICC(2) gauges absolute agreement, and is calculated using two-way ANOVA as follows: (MSB−Mean Squared Error within Evaluators (MSE))/(MSB + (Evaluators-1)×MSE + Evaluators×(Mean Squared sum of squares between Evaluators (MSJ)-MSE)/number of Participants). The addition of the k in ICC(1k) and ICC(2k) considers the means of four Evaluators. The interpretation of inter-rater agreement followed Fleiss’ suggestions: less than 0.40—poor, between 0.40 and 0.75—fair-to-good, and between 0.75 and 1.00—excellent [10] Normality of the distribution of continuous variables was tested using the Shapiro–Wilk test. Continuous variables were expressed as mean ± standard deviation (SD) or median (interquartile range, IQR), as appropriate. Statistical significance was set at *p* < 0.05. All analyses were completed with R Statistical Software (version 4.2.3, Foundation for Statistical Computing, Vienna, Austria).

## Results

**Fig. 1:**
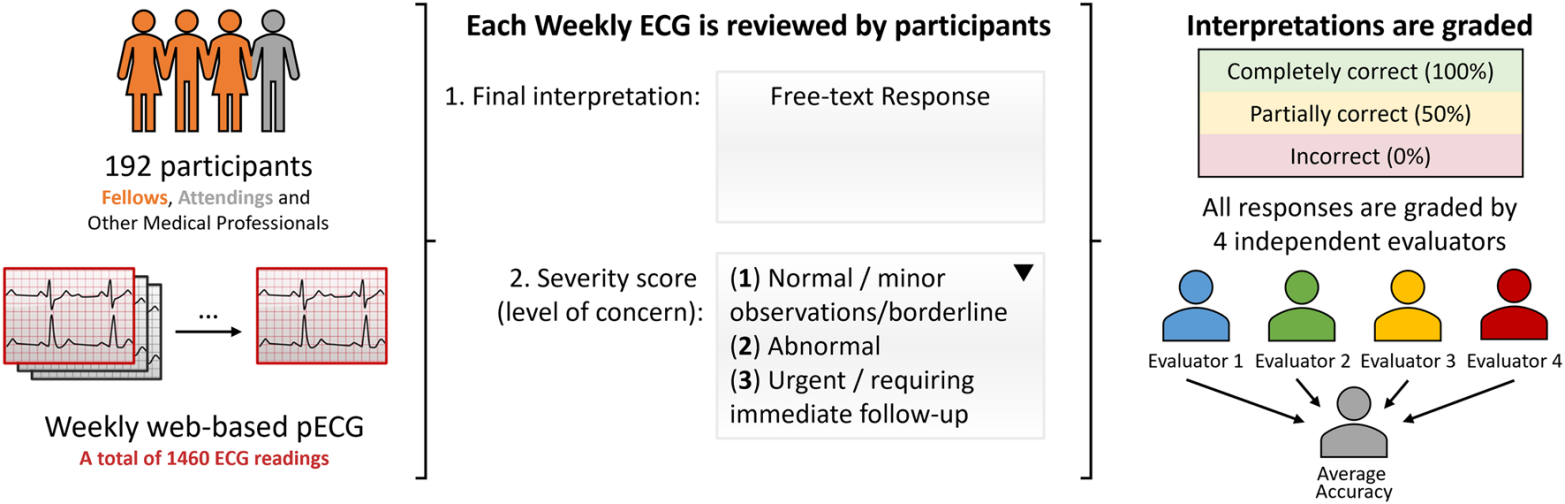
Study design and methodology

**Fig. 2:**
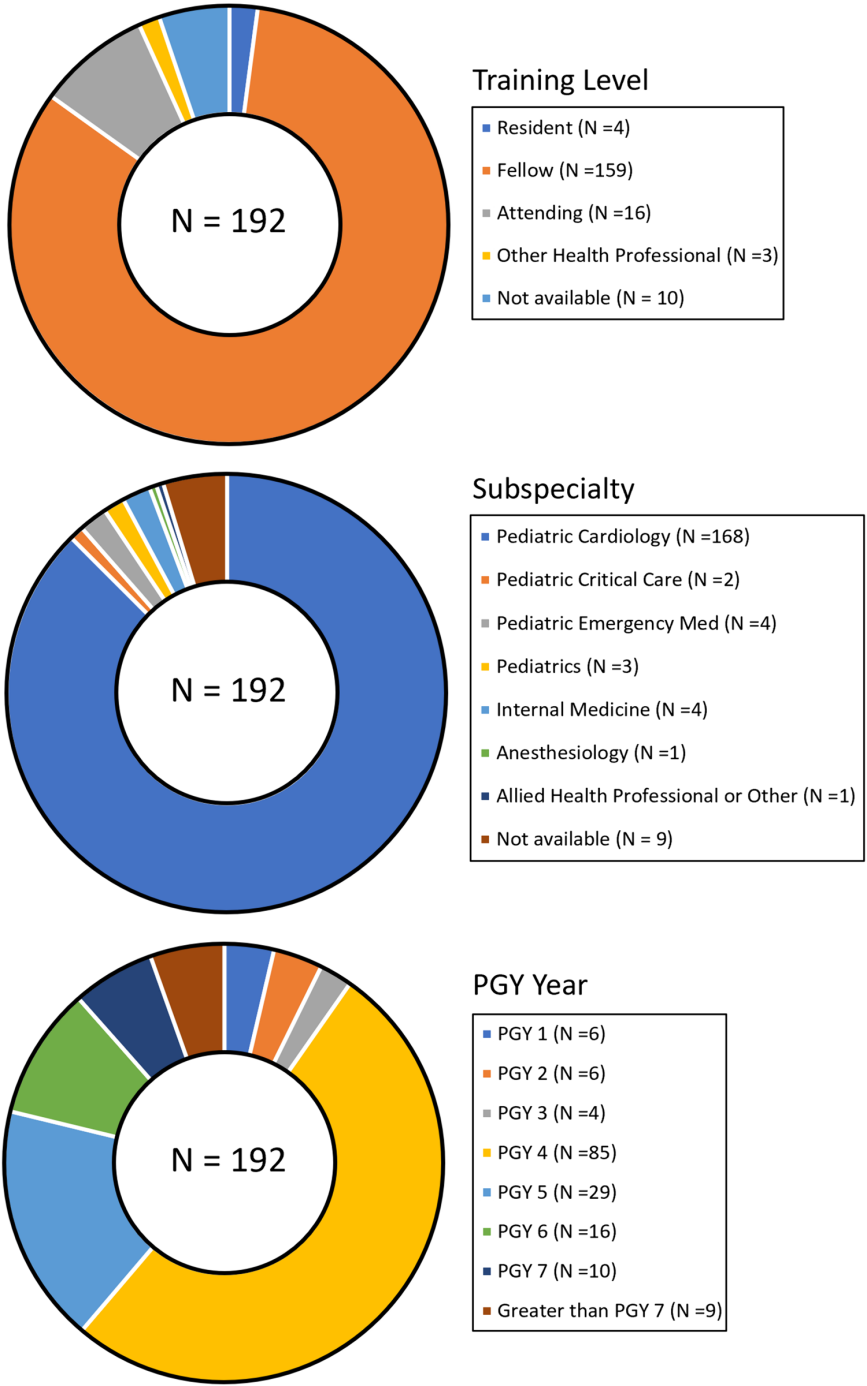
Participant demographics

One hundred ninety-two participants enrolled in the pECGreview program (Table 1, Fig. 1) and submitted 1460 completed responses. Participant level of training at registration, subspecialty and year of post-graduate training are presented in Fig. 2. Most participants were fellows in Pediatric Cardiology (78.1%), and the total number of responses per participant ranged from 1 to 19, with an average of 7.6 ± 6.2. The 19 ECGs were interpreted by an average of 76.8 ± 1.8 (range 59–92) participants although the group of participants for each ECG varied. Of all participants, 84 (44.3%) interpreted fewer than five ECGs. These were excluded from analysis of individual participant accuracy. The remaining 107 participants interpreted 1293 ECGs. The demographics of this group were similar to those of the entire population (Table 1). The four coders scored an Accuracy of 100% for 66.1 ± 5.1% of interpretations. An Accuracy score of 50% was assigned to 21.5 ± 4.6% of responses and an Accuracy score of 0% was assigned to 12.3 ± 3.9% of responses. The mean accuracy score of the 107 participants with five or more responses was 76.6 ± 13.7% (IQR 68.7–87.1%).

**Table 1:** Demographics for all participants and the subgroup which interpreted at least five ECGs

Characteristic	All participants (*N* = 192) Number (%)	≥ 5 ECGs (*N* = 107) Number (%)
Level of training at registration		
Resident	4 (2.08)	1 (0.93)
Fellow	159 (82.81)	88 (82.24)
Attending	16 (8.33)	11 (10.28)
Other Health Professional	3 (1.56)	2 (1.87)
Not available	10 (5.21)	5 (4.67)
Subspecialty		
Pediatric Cardiology	168 (87.5)	97 (90.65)
Pediatric Critical Care	2 (1.04)	1 (0.93)
Pediatric Emergency Med	4 (2.08)	2 (1.87)
Pediatrics	3 (1.56)	0 (0.00)
Internal Medicine	4 (2.08)	1 (0.93)
Anesthesiology	1 (0.52)	1 (0.93)
Allied Health Professional	1 (0.52)	0 (0.00)
Not available	9 (4.69)	5 (4.67)
Total number of responses	8.26 ± 6.90	13.11 ± 5.44
1–4	84 (43.75)	0 (0.00)
5–10	38 (19.79)	37 (34.58)
11–15	31 (16.15)	31 (28.97)
16–20	28 (14.58)	28 (26.17)
> 20	11 (5.73)	11 (10.28)

Table 2 demonstrates that there is a systematic difference in mean Accuracy score for Evaluator 2, one of the two electrophysiologists, whose scores tend to be lower, e.g., more critical, compared to the mean scores of the other three evaluators (ANOVA *F* = 7.20, *p* < 0.001). Nevertheless, the standard deviations remain relatively consistent across all evaluators at 19% of mean Accuracy score.

**Table 2:** Participant accuracy according to each evaluator

Evaluator	Accuracy score Mean (SD)
Evaluator 1	78 (14.3)
Evaluator 2	70.7 (16.4)
Evaluator 3	79.4 (14.5)
Evaluator 4	78.4 (14.1)

**Fig. 3:**
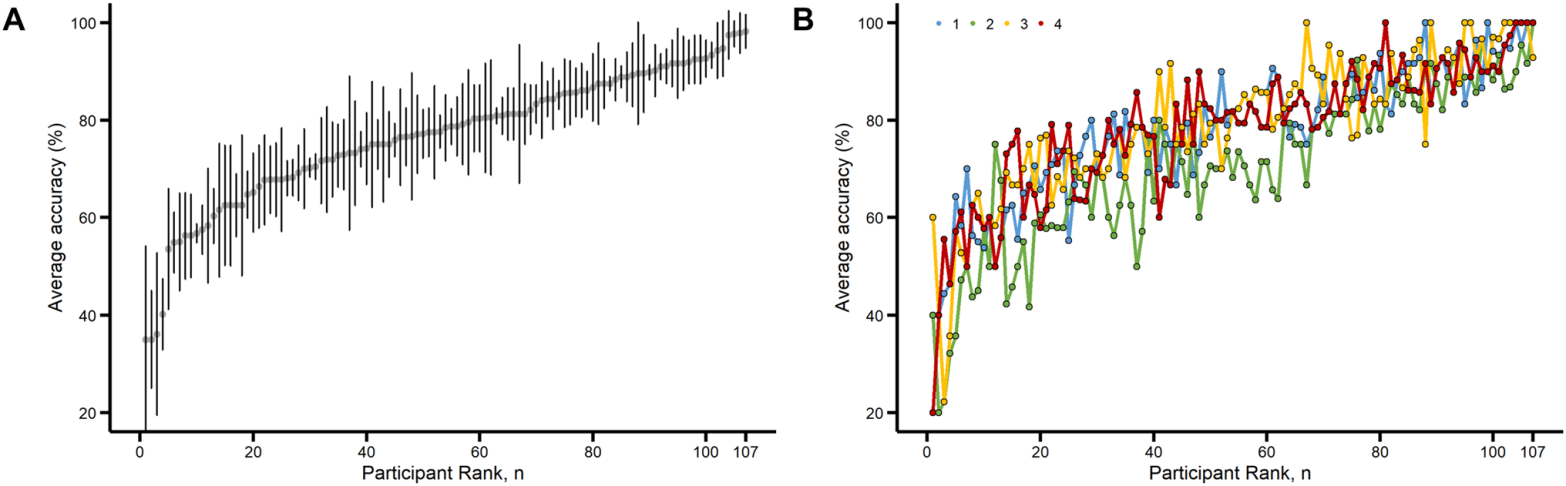
Accuracy score. Average Accuracy score for participants who interpreted at least five ECG is plotted in rank order. **A** Mean and standard deviation of scores for the four Evaluators. **B** Mean Accuracy score for each of the four Evaluators. Evaluator 2 (green markers) exhibited overall lower Accuracy scores than the other evaluators. The accuracy scores of the two EP evaluators are shown as the blue and green lines

**Fig. 4:**
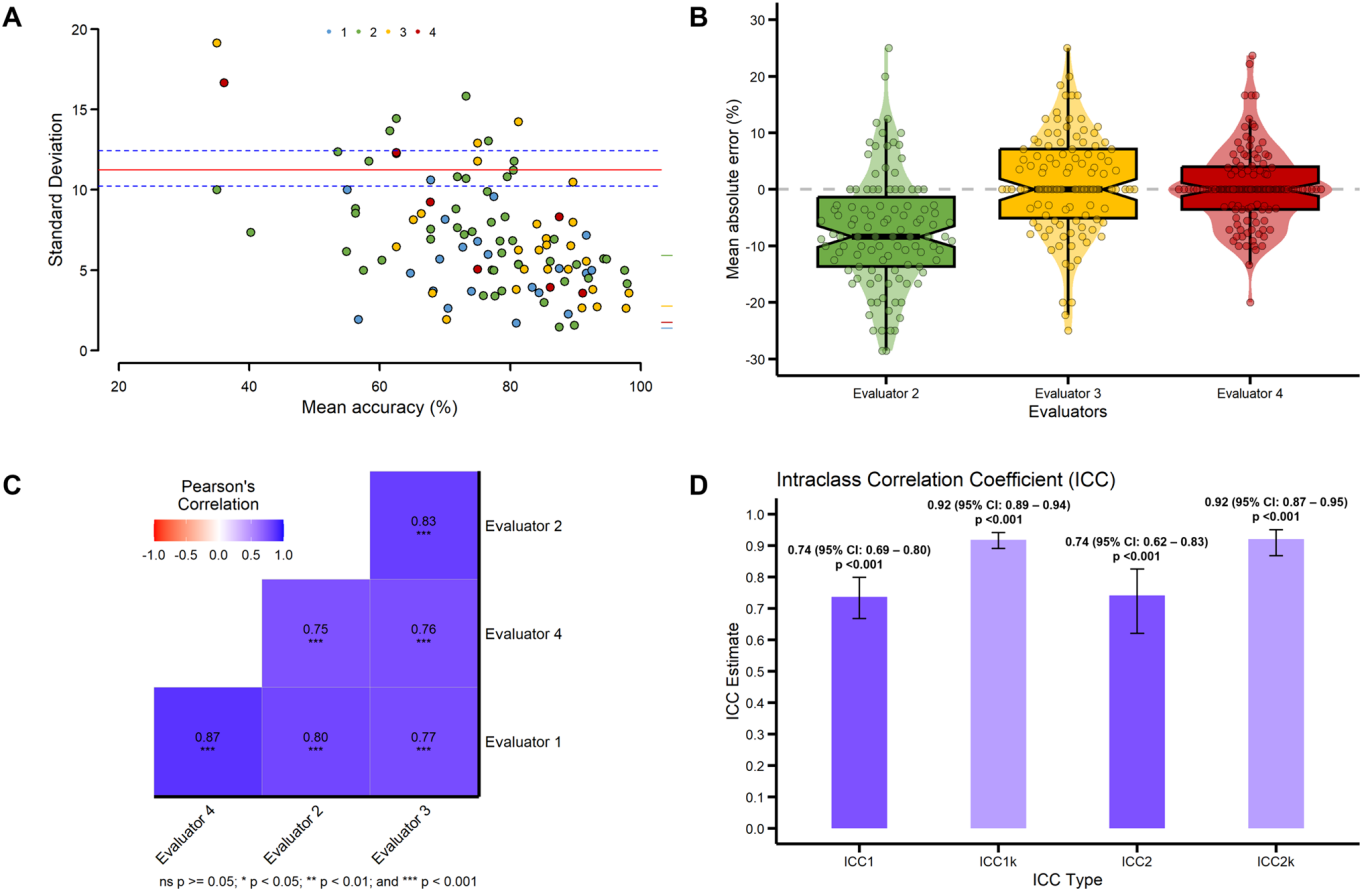
Validation and agreement of the Accuracy score. Validation of the Accuracy score reliability between four Evaluators by **A** extended Bland–Altman plot, **B** Mean Absolute Error, **C** Pearson’s Correlation, and **D** Intraclass Correlation Coefficient. See text for detailed interpretation

The mean Accuracy score was also calculated for each individual participant that interpreted at least five ECGs. This provides a relative ranking of the participants ECG interpretation skills. The mean Accuracy for each participant is visually represented in rank order in Fig. 3, both as mean and standard deviation of all scores (Fig. 3A) as well as the mean scores from each of the four independent evaluators (Fig. 3B). These figures demonstrate that the variability among participants is significantly greater than the variability within participants (e.g., across evaluators). The extended Bland–Altman plot (Fig. 4A) indicates a 95% LOA of 11.3 (95% bias-corrected LOA 10.3–12.4) for the Accuracy score. Furthermore, all the outlier measurements (above the red LOA line) occur for participants with lower mean accuracy. Thus, agreement between evaluators goes up as the average accuracy increases. We did observe some systemic bias, as indicated earlier, where the green tick mark representing Evaluator 2 (see to the right of the Fig. 4A plot) is elevated compared to the other three evaluators. Figure 4B demonstrates the source of the systemic bias for Evaluator 2 by comparing the mean absolute error for Evaluators 2, 3, and 4 as compared to Evaluator 1. Evaluator 2 had a tendency toward assigning lower Accuracy scores than the other Evaluators. However, pairwise inter-rater agreement was consistently good, with all correlations being equal to or above 0.75 and statistically significant, as depicted in Fig. 4C. As seen in Fig. 4D, intraclass correlation coefficients were all at the threshold between fair-to-good and excellent. Absolute agreement, ICC1, was 0.74 (95% CI 0.69–0.80), *p* < 0.001, and ICC2, 0.74 (95% CI 0.62–0.83), *p *< 0.001, respectively. Average measures agreement was excellent, ICC1k, was 0.92 (95% CI 0.89–0.94), *p* < 0.001, and ICC2k, 0.92 (95% CI 0.87–0.95), p < 0.001, respectively.

**Fig. 5:**
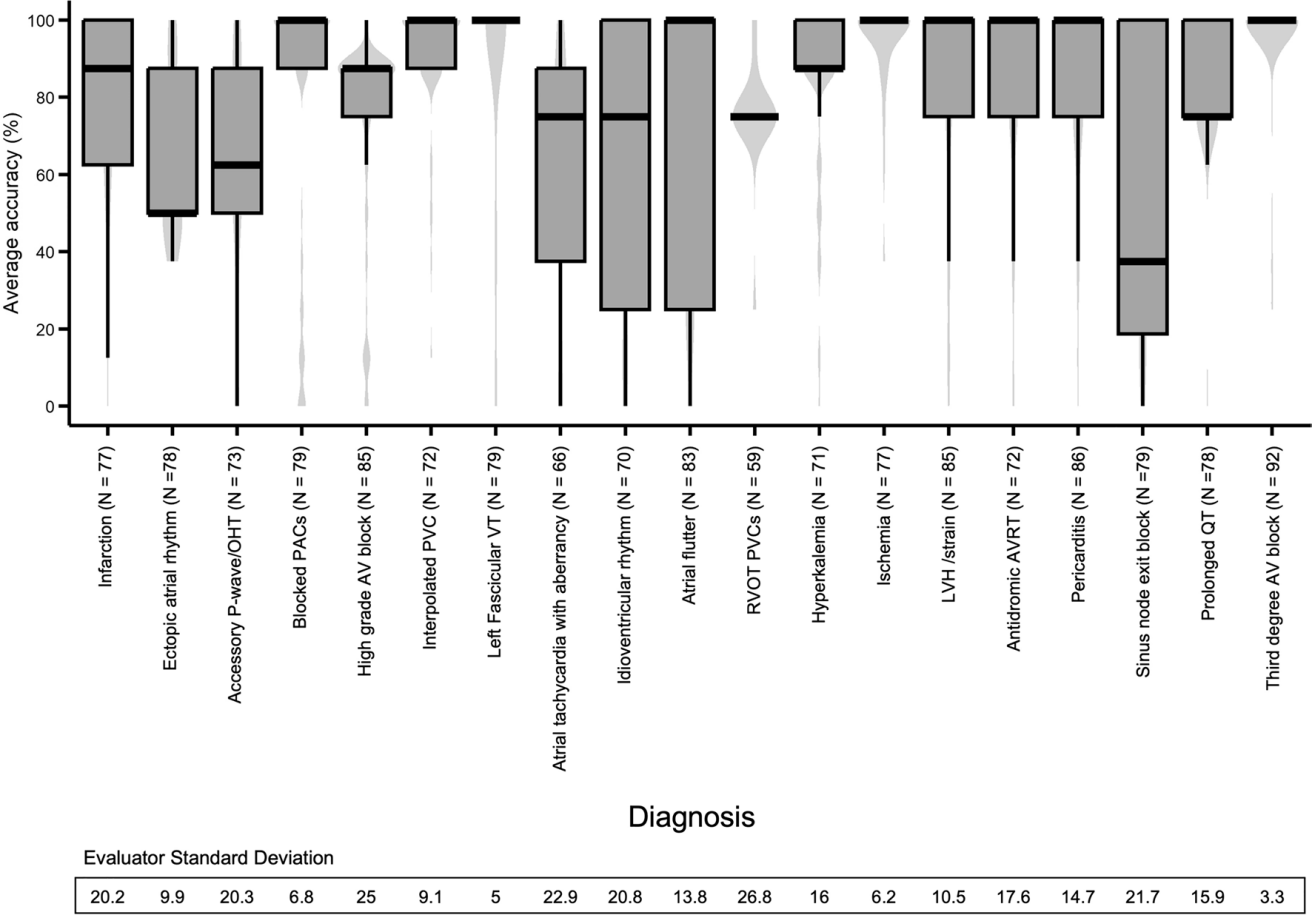
Average accuracy score stratified to ECG diagnosis. Validation of the overall Accuracy score stratified to ECG diagnosis, with evaluator standard deviation per diagnosis. *AV* atrioventricular, *AVRT* atrioventricular reentry tachycardia, *LVH* left ventricular hypertrophy, *OHT* orthotopic heart transplant, *PACs* premature atrial contractions, *PVC* premature ventricular contraction, *RVOT* right ventricular outflow tract, *VT* ventricular tachycardia

The Accuracy score (mean ± SD) of all participants as stratified according to each pECG, as well as the standard deviation of the four evaluators is reported in Fig. 5. There was a wide range in mean Accuracy and standard deviation between the various ECGs.

## Discussion

We present an investigation of the accuracy and consistency of pediatric ECG interpretations among pediatric cardiology fellows, trainees, and faculty enrolled in a large multicenter educational registry. The database provided by the pECGreview repository is the largest source of pediatric trainee ECG interpretations currently available and provides a resource for comparing multiple interpretations of the same ECG. For example, the 59 to 92 interpretations for each of the 19 ECGs in this cross-sectional study is larger than many previous studies of pediatric ECG interpretations which evaluated on average only 10 ECGs each. Longitudinal studies using the pECGreview database have the potential for providing interpretations of up to 156 ECGs during the course of a pediatric cardiology fellowship or other training program.

The primary finding of the current study was that the use of the Accuracy methodology provides the first, validated, quantitative means to assess the quality of ECG interpretations of individuals interpreting a series of standardized ECG images.

This study demonstrates between fair-to-good and excellent interrater agreement, validating the adequacy of our methods. Although there may be some variation in mean Accuracy scoring between evaluators, even single rater reliability was good, and the observed variation had little effect on ranking of individual participants with as few as five pECG interpretations. The primary difference among the evaluators was related to the one electrophysiologist who tended to score the interpretations “harder” by 8%. However, since this difference was consistent between the various participants, it effected the absolute scores but had little effect on the participant ranking. The finding that single rater reliability was good even between evaluators with widely different levels of training (ranging from medical student to experienced pediatric electrophysiologist) provides critical validation for the use of a single evaluator in future large scale longitudinal studies of pECGreview responses. The identified variations in evaluator scores do emphasize the need for standardized criteria and continuous quality improvement in pECG interpretation training.

Previous studies have used a range of methodologies to examine the accuracy of pECG interpretations in a variety of different clinical settings and training statuses but have consistently demonstrated an alarmingly low accuracy rate. One study, using a two-point scale for 17 ECGs, found that the accuracy rate was better for senior pediatric residents, 64.1%, in comparison to residents, scoring 55.0% on average [11] Two similar studies have used a three or four-point scale and 10 ECGs each to evaluate various populations. Among pediatric attendings the mean “knowledge score” ranged from 47.7 to 69.7% [2] For pediatric residents and attendings, Kayar et al., found that the “rate of correctly defining the distinction between normal and abnormal was 93.7%” but the “rate of detecting pathologies in ECGs accurately and correct identification of specific diagnosis was 56.7%” [12]. The accuracy rate in that study did not improve following an intervention [12].

Other approaches have also been reported. A study of six attending-level pediatricians and two nurse practitioners demonstrated that after educative programs, accuracy rates for ECG findings improved drastically, suggesting the potential of continued education [13]. However, the participants in that study evaluated the same series of 11 ECGs for both the pre- and post-test assessments. Another study evaluated the interpretation skills by having the participants match 10 ECGs to a list of 10 diagnosis [1]. This multiple-choice approach is limited in that it is a poor correlate to the clinical situation where the number of potential interpretations is large and must be developed by the interpreter.

We also report poor interpretation skills results as derived from the pECGreview database. The overall accuracy of responses for this cohort, primarily fellows in pediatric cardiology training programs, was limited although somewhat better than the results described above, with 66% scored as generally correct. In 22% of interpretations there was an under or over diagnosis of a minor ECG finding and in 12% there was under or over diagnosis of a major ECG finding. However, it is important to stress that direct comparisons of ECG interpretation accuracy between these studies are limited due to the wide range of participant training, ECGs diagnoses, and scoring methods.

Comparison of the accuracy of the responses between the 19 ECGs used in this study as depicted in Fig. 5 is primarily significant for the marked differences in both mean accuracy and the standard deviation of the accuracy responses. By observation is clear that some pECG tracings were found to be easier to interpret correctly than others. However, the variation in response accuracy does not appear to be a simple dependence on the ease of interpretation.

The current study also provides the first reported ranking of the interpretations skills of individual participants and may be a helpful tool for participants by providing objective feedback on their ECG interpretation skills. This approach also provides a framework for future descriptive and interventional studies to improve pediatric ECG interpretation skills for health care providers at all levels.

### Limitations

Our study’s strengths lie in the use of a multicenter registry, with a large number of participants and pECGs with multiple interpretations and a panel of evaluators for each ECG interpretation. One limitation is that participants in pECGreview are not required to submit interpretations, so the cohort of participants is variable for each ECG. In addition, the Accuracy score assessment is a subjective scoring system resulting in demonstrable systemic bias (see Fig. 4). However, our analysis demonstrates that this approach to Accuracy scoring is sufficiently robust to result in reliable data. This cross-sectional study was not designed or powered to address the effect of the length of time in a cardiology fellowship training program or the extent of participation in pECGreview on the Accuracy score. This could introduce bias, as participants may potentially lack confidence in their interpretation for a particular ECG and so only respond to ECG’s that they feel they know the correct interpretation. That would lead to an overestimation of the observed accuracy. Another limitation of the ECG-of-the-week format includes the testing of ECG interpretation outside of a true clinical context. Some ECG’s are presented without the aid of a computer-generated interpretation, a feature often available in clinical settings, which is known to enhance diagnostic accuracy [14]. However, previous research has shown that computer-assisted interpretations of pediatric ECGs can be in disagreement with pediatric cardiologists, particularly in rhythm diagnosis, and among others the recognition of right bundle branch block, right ventricular hypertrophy, and QT analysis [14].

## Conclusion

Our study demonstrates that the Accuracy score provides a robust and reliable mechanism for assessing the quality of pECG interpretations among pediatric cardiology fellows participating in pECGreview. The relatively low mean Accuracy score in this cohort reinforces the importance of addressing competency gaps in ECG interpretation through specialized training programs, such as the pECGreview initiative.
